# Scavenger Activity Evaluation of the Clove Bud Essential Oil (*Eugenia caryophyllus*) and Eugenol Derivatives Employing ABTS^+•^ Decolorization

**DOI:** 10.3797/scipharm.1109-11

**Published:** 2011-10-17

**Authors:** Diego R. Merchán Arenas, Amner Muñoz Acevedo, Leonor Y. Vargas Méndez, Vladimir V. Kouznetsov

**Affiliations:** 1 Laboratorio de Química Orgánica y Biomolecular, Escuela de Química, Universidad Industrial de Santander, A. A. 678, Bucaramanga, Colombia; 2 Departamento de Química y Biología, Universidad del Norte, A.A. 1569, Barranquilla, Colombia; 3 Grupo de Investigaciones Ambientales, Facultad de Química Ambiental, Universidad Santo Tomás, A. A. 1076, Bucaramanga, Colombia

**Keywords:** Clove essential oil, Eugenol, Isoeugenol, Antioxidant activity, ABTS radical-cation, GC-MS, Free radical scavenger activity

## Abstract

The essential oil (EO) of clove bud dried fruits from *Eugenia caryophyllus* was obtained by a conventional hydrodistillation process in an excellent yield (11.7 %). Its chemical composition was analyzed by GC-MS, identifying eugenol as a main constituent (60.5%). Four eugenol-like molecules, γ-diisoeugenol, hydroxymethyleugenol, dihydroeugenol and 1,3-dioxanylphenol, were synthesized using eugenol or isoeugenol as initial precursors under green chemistry protocols. To evaluate the possible antioxidant capacity of eugenol compounds including the clove bud EO, the Trolox® Equivalent Antioxidant Capacity value, obtained by the ABTS^+•^ radical-cation discoloration method, was employed. The methodology was performed in a UV-Vis reader of 96-well microplates (dilution methodology), using well-known antioxidant agents (BHA, BHT and vitamin E) as reference compounds. It was found that the prepared eugenol derivatives had a more potent free radical scavenger activity than the reference compounds. In particular, the most active molecules, γ-diisoeugenol and 1,3-dioxanylphenol, were *ca*. 3-fold more potent than vitamin E.

## Introduction

Many extensive studies on antioxidant agents and oxidative stress indicate their close relationship with aging [1]. External factors (cigarette smoking [2], excessive exercise [3–5] and the environment [6]) increasingly affect the cellular balance. The misbalance of excessive production of pro-oxidant agents or free radicals causes cell damage [7, 8]. Thus, the increase of reactive oxygen radical species (ROS, NO^•^, HO^•^, ROO^•^, O_2_^•−^) produced by mitochondria, the main organelle involved in the respiratory process, generates many structural changes to the primary metabolites [9–11] that cause many human diseases [12]. Diabetes [13], cardiovascular disease [14], carcinogenesis [15] and Alzheimer’s dementia [16, 17] are related to ROS generation. On the other hand, free radical oxidation of the lipid components in food due to the chain reaction of lipid peroxidation is a major strategic problem for food manufacturers [18]. Thus, there is an urgent need for the development of novel antioxidant agents, of both natural and synthetic origins.

**Fig. 1:**
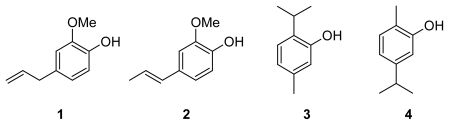
Natural phenols in essential oils

Natural products are emerging as the leading alternative to prevent some diseases produced by free radicals, either in humans, food and cosmetics. The best-known antioxidant agents are vitamin E (α-tocopherol), vitamin C (ascorbic acid) and several phenolic compounds found in food products. Plant or fruit extracts and essential oils (EO) also contain many different antioxidant molecules [19–23]. One of the oils used in foods because of its protective power of stabilizing free radicals is the EO of clove bud dried fruits [24]. Eugenol **1** together with isoeugenol **2**, thymol **3** and carvacrol **4** (Figure 1) are attractive molecular models in pharmacological and biological studies related to ROS inhibition [25].

Some synthetic phenolic compounds such as butylated hydroxy anisole (BHA), butylated hydroxytoluene (BHT), and tertiary butyl hydroquinone (TBHQ) have also been used as antioxidant agents to preserve a wide variety of foods. However, some studies indicated that these phenols, i.e. BHA, can affect liver and kidney functions [26].

Similarly, various methods are used to evaluate the antioxidant capacity of both natural and synthetic products. Among various methods for reactive species detection [27–29], the most popular methods are discoloration assays for stable radicals 1,1-diphenyl-2-picryl-hydrazyl (DPPH) [27] and 2,2′-azino-bis(3-ethylbenzothiazole-6-sulphonic acid) (ABTS) [28].

With these facts in mind and as a development of our medicinal program directed to small natural or synthetic molecules for drug delivery, we wanted to prepare and test new eugenol-like compounds as potential antioxidant agents. The purpose of this work was to synthesize four diverse eugenol-like molecules, γ-diisoeugenol, hydroxymethyleugenol, dihydroeugenol and 4-(1,3-dioxanyl)phenol and to evaluate its possible free radical scavenging capacity using the Trolox® Equivalent Antioxidant Capacity value, obtained by the ABTS^+•^ radical-cation discoloration method.

## Results and Discussion

The clove bud dried fruits were acquired at the local market. A conventional hydrodistillation process of this raw material provided the EO in a high yield (11.7 %). Chemical composition of isolated EO was determined by gas chromatography (DB-5, 30 m) coupled with mass selective detector (EI, 70 eV), employing as characterizing criteria the data system ChemStation G17001DA and its data base (NIST 2002, NBS 75K and WILEY 138K) that allowed identifying its principal components (Tab. 1).

According to reports [24], the major component in the clove oil corresponds to the eugenol. In our research, the EO contains 60.5 % of this phenolic compound with interesting pharmacological features, particularly antioxidant activity, and chemical moieties easily transformable.

Thus, looking for new eugenol-based molecules as potential free radical scavengers, we modified the structure of the eugenol **1** and its isomer, the isoeugenol **2**. The first modification was towards preparation of the dihydroeugenol **5**, another natural phenolic compound. This molecule is present in low quantities in the leaf oils from *Eucalyptus globulus* [30] and *Juniperus drupacea* L. [31]. The reduction of commercial eugenol was carried out using Pd/C (5 % mol) in 5 mL de PEG 400 as a new reaction media for the catalytic hydrogenation, which allowed obtaining product **5** in excellent yields. Its structure was easily confirmed by NMR data.

Then, we could introduce a hydroxymethyl fragment in the ring of **1** via Lederer-Manasse reaction [32, 33] using formaldehyde in methanol under basic conditions to give molecule **6** (Sch.1). Its ^1^H NMR spectrum showed CH_2_ protons signals and broad singlet of the non-aromatic OH of the hydroxymethyl moiety at 4.70 and 2.70 ppm, respectively, which confirmed molecular structure of obtained compound **6**. This reaction could be considered as an aromatic nucleophilic substitution reaction, S_N_2_Ar_.

**Fig. 2:**
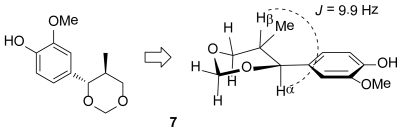
Stereochemistry of 2-methoxy-4-(5-methyl-1,3-dioxan-4-yl)phenol **7**

In the same way, the isoeugenol was exposed to the Lederer-Manasse reaction under basic conditions. However, according to the GC-MS and NMR experiments, the final product did not result in a similar molecule as **6**, but in a 4-(1,3-dioxanyl)phenol **7**, a Prins reaction-like product (Sch. 1). Some analogues of this derivative have been synthesized through the Prins reaction that is usually the acid-catalyzed addition of aldehydes to alkenes [33, 34]. In our case, formation of the 2-methoxy-4-(5-methyl-1,3-dioxan-4-yl)phenol **7** was promoted by a NaOH solution that was observed for the first time. The signals of Hα and Hβ in ^1^H NMR allowed us to define the stereochemistry of obtained molecule **7**. One doublet of Hα at 4.04 ppm and the coupling constant with high values close to 9.9 Hz, characteristic for axial-axial H interactions, confirmed strongly its stereochemistry (Fig. 2).

Finally, we prepared the diisoeugenol **8** with γ-configuration through a formal [3+2] cycloaddition reaction of the isoeugenol **2,** employing eco-friendly tools [35]. This compound has been used as an antioxidant agent in the protection of perfumes and some cosmetic products [36]. In spite of its important feature, its antioxidant action as a free radical scavenger was never reported. Its stereochemistry was studied employing 1D and 2D NMR experiments that indicated a γ-configuration, e.g. *r*-1-ethyl-5-hydroxy-*c*-3-(4-hydroxy-5-methoxyphenyl)-6-methoxy-*t*-2-methylindane **8**. All signals for the non-aromatic protons showed information about stereochemistry, the doublet signal observed for the 3-H, appear to 3.73 ppm with a constant coupling value of 9.5 Hz, characteristic for the antitype conformation of this system.

### Free radical scavenging capacity

With the easy preparation of the essential oil of clove bud and the eugenol derivatives, and based on literature bio information, we supposed that these compounds could act as free radical scavengers. Each one of these derivatives possesses phenolic hydroxyl and methoxy groups in ortho-position, an important moiety responsible for free radical scavenging activity. To evaluate the possible antioxidant capacity of eugenol compounds **5**–**8** including the clove bud EO, the Trolox® Equivalent Antioxidant Capacity (TEAC) value, obtained by the ABTS^+•^ radical-cation discoloration method, was employed [27]. The Trolox® equivalent antioxidant capacity assay is a wellknown method used to estimate an antioxidant substance ability to reduce the ABTS^+•^ radical-cation. In this assay, an antioxidant was added to a solution pre-formed out of the ABTS^+•^ radical-cation, and, within a fixed range of time, the ABTS^+•^ residual radical-cation was spectro-photometrically quantified. Thus, using own simple procedure in 96-well multiplate reader for radical scavenging capacity (reductive capacity), based on Trolox® equivalent antioxidant capacity (TEAC) [37] the interesting data for tested compounds were obtained. The clove bud EO TAA (Total antioxidant activity, mmol Trolox®/kg of EO) was also calculated (Tab. 2).

All TEAC values obtained for the eugenol derivatives **5**–**8** were higher than that of the commercial antioxidants (vitamin E, BHT and BHA). It was noted that dihydroeugenol **5** resulted less active scavenger than its synthetic precursor **1,** while the introduction of another hydroxyl group like a hydroxymethyl moiety (CH_2_OH) in its structure (comp. **6**) enhanced antioxidant capacity. Scavenger activity of the hydroxymethyleugenol **6** was evaluated employing DPPH method [25], and our modified ABTS^+•^-methodology [36] has showed a good similarity. The most active molecules, 4-(1,3-dioxanyl)phenol **7** and γ-diisoeugenol **8**, were ca. 3-fold more potent than the well-known antioxidant vitamin E. All tested compounds revealed a prominent anti-radical capacity, but compounds, in descending order, **8** > **7** > **6** > **1** > **5** showed a higher activity than the commercial antioxidants (α-tocopherol, BHT and BHA). It is known that polimeric or dimeric phenols (comp. **8**) have a higher antioxidant (antiradical) activity than monophenols (comp. **1**, **5–7**). Comp. **7** could be considered as a monophenol para-substitued with a strong electron donating group like 1,3-dioxanyl. According to published report [38], the high scavenger capacity of these derivatives is generally attributed to the phenolic functional group; however, these tested monophenols with ortho-methoxy group could act with free radicals through different mechanisms after the abstraction of the hydrogen atom from the phenolic species ArOH to phenoxy radical ArO^•^: The formed phenoxy radical can donate a second hydrogen, following by the electron delocalization into the para-substituted group, or dimerize between two phenoxy radical units. From the results obtained in the present study, it is evident that the monophenols-ABTS^+•^ interaction of these molecules will depend on the chemical nature of the para-substituents. For example, the phenols **1** and **6**, with the para-propenyl group, were more active than the phenol **5** with the propyl chain, indicating a possible mechanism in which a possible dimerization of **5** occurs [39]. For the diphenol **8**, the mechanism could be more complex.

In summary, we have employed an efficient and simple procedure in a 96-well multiplate reader for radical-scavenging capacity (reductive capacity), based on TEAC (Trolox® equivalent antioxidant capacity) assay that allowed testing both natural and synthetic substances, e.g., eugenol derivatives (which have relevant structural moieties like a methoxy group) and the clove bud EO. The reinvestigation of the γ-diisoeugenol **8** confirmed strongly its free radical scavenging capacity using TEAC assay. Antioxidant properties and easy preparation of novel 4-(1,3-dioxanyl)phenol **7** make it attractive as a molecular model in pharmacological research.

## Experimental

The precursors were of synthesis degree, and they were used without previous purification. The melting points (uncorrected) were determined on a Fisher-Johns melting point apparatus. The IR spectra were recorded on a Lumex Infralum FT-02 spectrophotometer in KBr and thin film. ^1^H NMR spectra were recorded on Bruker AM-400 spectrometer. Chemical shifts are reported in ppm (δ) relative to the solvent peak (CHCl_3_ in CDCl_3_ at 7.24 ppm for protons). Signals are designated as follows: s, singlet; d, doublet; dd, doublet of doublets; ddd, doublet of doublets of doublets; t, triplet; dt, doublet of triplets; td, triplet of doublets; q, quartet; quint., quintet; m, multiplet; br, broad. A Agilent Technologies 6890 plus Gas Chromatograph interfaced to an Agilent Technologies MSD 5963 Selective Detector (MSD) with a ChemStation Data system G17001DA was used for MS identification at 70 eV using a 60 m capillary column coated with HP-5 [5 %-phenyl-poly(dimethyl-siloxane)]. Elemental analyses were performed on a Perkin Elmer 2400 Series II analyzer and were within ± 0.4 of theoretical values. The reaction progress was monitored using thin layer chromatography on a silufol UV254 TLC aluminum sheet.

### Hydrodistillation of clove bud dried fruits for the essential oil

The essential oil from clove bud dried fruits of *Eugenia caryophyllus* acquired at the local market was isolated with a conventional hydrodistillation process. The Clevenger assembly with a Dean-Stark trap was used. 236.08 g of raw material were used, and it was kept to boiling water (200 mL) for 8 h. The extraction yield was 11.7 %. It was collected in amber vial with Na_2_SO_4_. The principal components of EO were characterized by gas chromatography with mass selective detector, employing as characterizing criteria the data system ChemStation G17001DA and its data base (NIST 2002, NBS 75K and WILEY 138K).

### Gas Chromatography–Mass Spectrometry (GC-MS) Analysis

GC-MS analyses was performed using HP-5 % phenyl-polymethoxylsiloxane column like a stationary phase DB-5MS, 30 m × 0.25 mm i.d., film thickness = 0.25 μm. Helium (99.9995%, Aga Fano, S. A.) was used as carrier gas with de 35 cm/s lineal velocity. The oven temperature was programed: 45 °C (5 min), @ 4 °C/min until 150 °C (2 min), @ 5 °C/min, until 250 °C (5 min), @ 10 °C/min, until 275 °C (5 min). The total run time was 17 min. The temperature ionization chamber and the transfer line were 230 y 285 °C, respectively. The injection volume was 2 μ with 1:30 split relation and the entry pressure on column was 15 psi. The ionization energy was 70 eV, and mass range was 40–400 m/z.

### Preparation of eugenol derivatives

#### 2-Methoxy-4-propylphenol (**5**)

Eugenol **1** (1.00 g, 6.1 mmol) and Pd/C (0.06 g) were mixed in 5 mL of PEG 400 as reaction medium. The hydrogen purge was established, and the reaction was allowed stirring overnight at room temperature. The reaction mixture was filtered and extracted with CH_2_Cl_2_ (2 × 10 mL). The resulting crude product was purified by chromatography column to give the dihydroeugenol **5**, 1.80 g (90 %) as colourless oil. IR (thin film): 3445 cm^−1. 1^H NMR (400 MHz, CDCl_3_), δ (ppm): 0.94–0.99 (3H, t, *J* = 7.32 Hz, -CH_3_), 1.58–1.59 (2H, sextet, -βCH_2_), 2.51–2.59 (2H, t, *J* = 7.5 Hz, -αCH_2_), 3.89 (3H, s, -OCH_3_), 5.54 (1H, s, -OH), 6.70 (1H, d, *J* = 7.5 Hz, 5-H), 6.71 (1H, s, 3-H), 6.86 (1H, d, *J* = 7.9 Hz, 6-H). MS (EI) m/z (relative intensity): 166 (M^+•^, 20), 137 (100), 122 (10). Elemental analysis: found: C, 64.45; H, 6.41. Calc. for C_10_H_14_O_2_: C, 64.68; H, 6.24 %.

#### 4-Allyl-2-(hydroxymethyl)-6-methoxyphenol (**6**)

Eugenol **1** (1.00 g, 12.2 mmol) were mixed with a NaOH solution (8 % wt), prepared from NaOH (0.98 g, 24.4 mmol) of in 13.0 mL of H_2_O. Then, 2.0 mL of CH_2_O/MeOH (35 %) were added; the reaction mixture was stirred at 50 °C for 5 h (TLC). The resulting mixture was neutralized with 20 % acetic acid (to pH 5) and extracted with CH_2_Cl_2_ (2 × 20 mL). The combined extracts were dried with sodium sulfate, concentrate to vacuum and purified by chromatography column to yield the hydroxymethyleugenol **6**, 0.54 g (54 %) as yellow oil. IR (thin film): 3436, 1150 cm^− 1. 1^H NMR (400 MHz, CDCl_3_), δ (ppm): 2.70 (1H, s, OH), 3.31 (2H, d, *J* = 6.7 Hz, γCH_2_), 3.86 (3H, s, COCH_3_), 4.70 (2H, s, CH_2_), 5.06 (2H, m, αCH), 5.93 (1H, m, βCH), 6.17 (1H, s, OH), 6.65 (1H, br. s, 3-H), 6.66 (1H, br. s, 5-H). ^13^C NMR (100 MHz, CDCl_3_), δ (ppm): 146.4, 141.9, 137.6, 131.4, 120.6, 115.5, 71.6, 61.7, 56.0, 39.8, 31.6. MS (IE) m/z (relative intensity): 194 (M^+•^, 40), 176 (100), 147 (50). Elemental analysis: found: C, 68.15; H, 6.99. Calc. for C_11_H_14_O_3_: C, 68.02; H, 7.27 %.

#### 2-Methoxy-4-[(4S,5S)-5-methyl-1,3-dioxan-4-yl]phenol (**7**)

Isoeugenol **2** (1.00 g, 12.2 mmol) were mixed with a NaOH solution (8 % wt), prepared from NaOH (0.98 g, 24.4 mmol) of in 13.0 mL of H_2_O. Then, 2.0 mL of CH_2_O/MeOH (35 %) were added; the reaction mixture was 50 °C and stirred during 5 h (TLC). The resulting mixture was neutralized with 20 % acetic acid (to pH 5) and extracted with CH_2_Cl_2_ (2 × 20 mL). The combined extracts were dried with sodium sulphate, concentrate to vacuum and purified by chromatography column to give 2-methoxy-4-(5-methyl-1,3-dioxan-4-yl)phenol **7**, 0.57 g (57 %) as colourless oil. IR (thin film): 3435, 1150 cm^−1. 1^H NMR (400 MHz, CDCl_3_), δ (ppm): 0.59 (3H, d, *J* = 6.7 Hz, CH_3_), 2.02–2.16 (1H, m, βCH), 3.38 (2H, ‘t’, *J* = 11.2 Hz 3′-H), 3.89 (3H, s, OMe), 4.04 (1H, d, *J* = 9.9 Hz, αCH), 4.09–4.13 (1H, dd, *J* =11.5, 4.5 Hz, 3′-H), 4.82 (1H, d, *J* = 6.3 Hz, 2′-H), 5.19 (1H, d, *J* = 6.3 Hz, 2′-H), 6.80 (1H, dd, *J* = 8.1, 1.6 Hz, 5-H), 6.89 (1H, d, *J* = 8.1 Hz, 6-H), 6.90 (1H, d, *J* = 1.6 Hz, 3-H). ^13^C NMR (100 MHz, CDCl_3_), δ (ppm): 146.7, 145.6, 131.5, 120.7, 114.3, 113.8, 109.3, 94.1, 86.0, 72.9, 55.9, 36.4. MS (EI) m/z, (relative intensity): 224 (M^+•^, 10), 152 (100), 137 (5). Elemental analysis: found: C, 64.43; H, 7.28. Calc. for C_12_H_16_O_4_: C, 64.27; H, 7.19 %.

#### *r-1-Ethyl-5-hydroxy-cis-3-(4-hydroxy-5-methoxyphenyl)-6-methoxy-trans-2-methylindane (rel-(1*R*,2*R*,3*R)*-1-Ethyl-3-(4-hydroxy-3-methoxyphenyl)-6-methoxy-2-methyl-2,3-dihydro-1*H*-Inden-5-Ol,* **8**)

To *trans*-isoeugenol **2** (1.00 g, 6.10 mmol) dissolved in PEG-400 (5 mL) was added 0.086 g of BF_3_•OEt_2_ (10 % mmol) at 0 °C. The reaction mixture was taken slowly to room temperature and continuous stirring was maintained, and after complete conversion, as indicated by TLC. The reaction mixture was supported in silica gel and purified by chromatography column to obtain the respective, γ-diisoeugenol **8**, 0.65 g (65 %) as solid, mp 184–185 °C [29]. IR (KBr): 3487, 2962, 1265 cm^−1. 1^H NMR (400 MHz, CDCl_3_), δ (ppm): 0.97 (3H, t, *J* = 7.3 Hz, Me), 1.03 (3H, d, *J* = 6.9 Hz, 2-Me), 1.31–1.44 (1H, m, CH_2_), 1.65–1.75 (1H, m, CH_2_), 2.40–2.51 (1H, m, 2-H), 2.85–2.95 (1H, m, 1-H), 3.73 (1H, d, *J* = 9.5 Hz, 3-H) 3.80 (3H, s, CH_3_O-Ar), 3.89 (3H, s, 6-OCH_3_), 5.51 (1H, s, HO-Ar), 5.56 (1H, s, 5-OH), 6.48 (1H, s, 7-H), 6.62 (1H, br. s, 2-HAr), 6.65 (1H, br. d, *J* = 8.0, 6-HAr), 6.77 (1H, br. s, 4-H), 6.62 (1H, d, *J* = 8.0, 5-HAr); ^13^C NMR (100 MHz, CDCl_3_), δ (ppm): 146.4, 145.1, 144.5, 144.1, 139.1, 138.7, 135.8, 121.5, 114.0, 111.0, 110.6, 107.5, 56.7, 56.1, 55.9, 49.2, 48.5, 22.4, 13.8, 12.2. MS: m/z (relative intensity): 328 (M^+•^, 60 %), 299 (100), 204 (40), 175 (25). Elemental analysis: found: C, 81.17; H, 8.03. Calc. for C_20_H_24_O_2_: C, 81.04; H, 8.16 %.

### Free-Radical Scavenging Activity (ABTS Assay)

The free radical scavenging activity was performed employing modified Re’ procedure [27, 37]. The optimization of ABTS^+•^ radical-cation was carried out with 3.34 mg of potassium peroxodisulphate (K_2_S_2_O_8_) and 19.60 mg of ABTS. This mixture was flasked with 5 mL HPLC water. The reaction was kept during 16 h between 10–15 °C without light. Then, an aliquot of ABTS^+•^ was diluted in absolute EtOH to obtain 0.700 of absorbance to 734 nm. Standard 1×10^− 3^ M samples solutions were analysed and then, were diluted again until that in the presence of ABTS^+•^ (200 μL). Its inhibition was between 20–80 % of the blank absorbance. The inhibition was evaluated after 30 min and was plotted in function of the concentrations established. All of the assays were performed in triplicate. The TEAC value was determined as the relationship between the 50% inhibitory concentrations (IC50) of Trolox® and the antioxidant in question.

## Figures and Tables

**Fig. 3:**
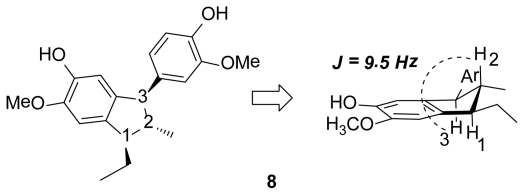
γ-Configuration of diisoeugenol **8**

**Sch. 1:**
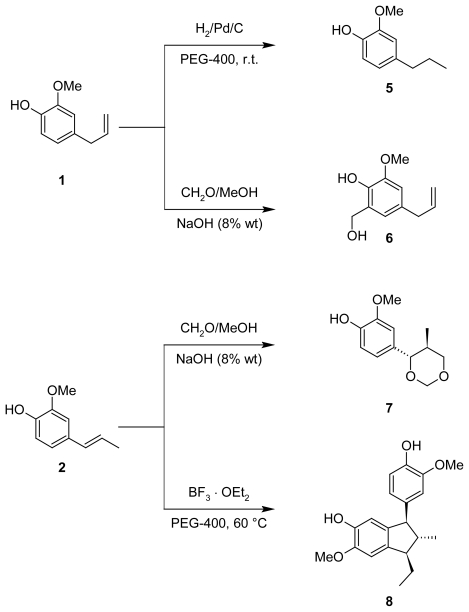
Synthetic routes to eugenol-based compounds

**Tab. 1:** Principal constituents in the essential oil of clove bud dried fruits from *Eugenia caryophyllus*

Compound	tR^a^ (min)	IR^b^	Percent^c^
Eugenol	34.35	1363	60.5
β-caryophyllene	37.09	1438	7.5
α-humulene	38.26	1472	1.7
Eugenyl acetate	39.82	1520	7.0

a Retention time on DB-5 column;

b Kovats Retention Indices;

c Percents are reported as gas chromatography areas without correction factor.

**Tab. 2:** TEAC values for the eugenol derivatives and TAA values for the clove bud EO and “control” substances

Sample	TEAC*	
	Average ± s	RSD (%)
Clove bud EO	(11900 ± 164)†	0.5
Eugenol **1**	1.68 ± 0.05	3
Dihydroeugenol **5**	1.59 ± 0.02	4
Hydroxymethyleugenol **6**	1.8 ± 0.1	4
4-(1,3-Dioxanyl)phenol **7**	2.49 ± 0.05	1
γ-Diisoeugenol **8**	2.61 ± 0.09	0.6
BHT	1.29 ± 0.04 (21000 ± 308)†	3
BHA	1.02 ± 0.04 (8000 ± 184)†	4
Vitamin E	0.89 ± 0.01 (3300 ± 47)†	1

* RSD %: Relative standard deviation;

* n = 3;

† Total antioxidant activity (mmol Trolox®/kg of EO).
